# Notes and News

**Published:** 1906-03-17

**Authors:** 


### Notes and News.

Under the will of the late Mr. Thomas Marbutt, of Edgbaston, the sum of £1,250 is bequeathed to the General Hospital, Birmingham, for the purpose of establishing a "Thomas Marbutt Bed." Smaller bequests are left to other local charities.

In reply to adverse critics of the Bill which has been introduced by Dr. Macnamara to prohibit juvenile smoking, the hon. member points out that the measure does not forbid the sale of tobacco in any shops to grown-up men, but is entirely limited in its application to punishment, on conviction, of the person who supplies it for the use of any persons under sixteen years of age.

The Duchess of Connaught will open the Elizabethan Fair and Fête, to be held at Lincoln's Inn Hall and Gardens on May 23 and 24 on behalf of the King's College Hospital Removal Fund. To this fund the directors of Harrods, Limited, have made a donation of 2,000 guineas, and by their wish the money will be placed to the credit of Lady Methuen's stall, and will eventually be applied to the founding of two beds in the new building.

At Oxford last week Dr. Osler presided over a large meeting which assembled in order to discuss the question of infant mortality and how it might be remedied. Dr. Moore, medical officer of health for Huddersfield, who was the chief speaker, insisted that in England at the present time there is going on in our midst a loss of life which might be designated constructive murder by the State. He held that if a responsible power permitted death to occur from causes which were indicated, and if these causes were readily preventible, it was no exaggeration to call it constructive murder. He urged the immense importance of the systematic teaching of hygiene and of the functions and duties of motherhood.

In speaking at a conference held at the Sanitary Institute last Tuesday, Mr. J. Russell, headmaster of Hampstead School, declared that part of the training of the teacher of the future must be in medical knowledge. Such an opinion has constantly been urged in the medical press, and it is encouraging to observe that its importance is beginning to be appreciated by those who are more immediately concerned.

In an address delivered at the Kaiser Wilhelms Akademie last week Professor Koch said that his studies had entirely confirmed the results of the investigations of Dr. Castellani and Dr. Bruce, and he had devoted his efforts in particular to investigating the habits of the *glossina palpalis*, the fly by which the infection of sleeping sickness is conveyed. He had found the fly chiefly on the shores of Victoria Nyanza, where it infested a certain tree, and was a parasite of birds.

A recent report of the Gold Coast Colony shows an astonishingly large increase in the criminal returns over those of the previous year. This is, to a great extent, accounted for by the increase of offences at Kumasi under the sanitary laws. Kumasi, together with some of the other principal towns of the colony, notably Cape Coast, Sekondi, and Axim, has been undergoing extensive sanitary improvements during the last two years, and the native, not content with "passive resistance," has in most cases put himself in active rebellion against this interference with his primeval habits. He was quite "content with the conditions under which his race had lived for centuries, and did not wish anything better." This excess of feeling is now beginning to abate a little, but it will be long before it is stamped out.

Last year we published some correspondence on the curious anomaly of hospital attendants being regarded as male servants by the Inland Revenue authorities, who on this ground claim the right to demand the payment of the usual license duty. The question is now raised again by a letter which we have received from a hospital secretary. This gentleman claimed exemption from the tax, on the ground that the hospital was a charitable institution. The Commissioners of Inland Revenue replied through their local officer that "there is no statutory exemption in respect of male servants employed in attendance on the sick poor, and that duty must therefore be paid in respect of the man in question." If this represents the final verdict of Somerset House, we think that the sooner the matter is put to the test of a legal decision the better, for although the imposition is a small one, it is none the less unjust and unreasonable, and runs counter to the principles upon which such license duties were originally levied. But we feel confident that if a direct appeal were made to the Inland Revenue authorities at Somerset House the claim for license duty would be withdrawn; and this is the course which we recommend to our correspondent and others who are faced with the same difficulty.

